# Amphotericin B in the Era of New Antifungals: Where Will It Stand?

**DOI:** 10.3390/jof10040278

**Published:** 2024-04-10

**Authors:** Karolina Akinosoglou, Emmanouil Angelos Rigopoulos, Despoina Papageorgiou, Georgios Schinas, Eleni Polyzou, Effrosyni Dimopoulou, Charalambos Gogos, George Dimopoulos

**Affiliations:** 1 School of Medicine, University of Patras, 26504 Patras, Greece; agrigopoulos@gmail.com (E.A.R.); dspn.pap96@gmail.com (D.P.); georg.schinas@gmail.com (G.S.); polyzou.el@gmail.com (E.P.); cgogos@upatras.gr (C.G.); 2 Department of Internal Medicine and Infectious Diseases, University General Hospital of Patras, 26504 Rio, Greece; 3 Hellenic Institute for the Study of Sepsis, 11528 Athens, Greece; efidimop98@gmail.com; 4 3rd Department of Critical Care, Evgenidio Hospital, Medical School, National and Kapodistrian University of Athens, 11528 Athens, Greece; gdimop@med.uoa.gr

**Keywords:** amphotericin B, mycoses, fungal infections, drug resistance, fungal, liposomes, antifungal agents, prophylactic treatment, immunocompromised host, epidemiology, fungal

## Abstract

Amphotericin B (AmB) has long stood as a cornerstone in the treatment of invasive fungal infections (IFIs), especially among immunocompromised patients. However, the landscape of antifungal therapy is evolving. New antifungal agents, boasting novel mechanisms of action and better safety profiles, are entering the scene, presenting alternatives to AmB’s traditional dominance. This shift, prompted by an increase in the incidence of IFIs, the growing demographic of immunocompromised individuals, and changing patterns of fungal resistance, underscores the continuous need for effective treatments. Despite these challenges, AmB’s broad efficacy and low resistance rates maintain its essential status in antifungal therapy. Innovations in AmB formulations, such as lipid complexes and liposomal delivery systems, have significantly mitigated its notorious nephrotoxicity and infusion-related reactions, thereby enhancing its clinical utility. Moreover, AmB’s efficacy in treating severe and rare fungal infections and its pivotal role as prophylaxis in high-risk settings highlight its value and ongoing relevance. This review examines AmB’s standing amidst the ever-changing antifungal landscape, focusing on its enduring significance in current clinical practice and exploring its potential future therapeutic adaptations.

## 1. Introduction

Over the past two decades, there has been a notable rise in the population of immunocompromised individuals who are vulnerable to developing invasive fungal infections (IFIs). This period has also witnessed a shift in fungal epidemiology, marked by an increase in infections caused by non-Aspergillus molds and yeasts. These particular fungi often exhibit resistance to one or more antifungal medications. Conventional diagnostic approaches, such as culture and histopathological examination of infected tissue, frequently fall short of detecting IFIs, especially in their early stages. Furthermore, invasive diagnostic procedures to obtain tissue samples may not be suitable for severely ill patients. Even when tissue samples are obtained, the morphology of various filamentous fungi can be indistinguishable, or the cultures may fail to cultivate the pathogen. Rapid diagnosis of invasive fungal infection is challenging, can have a long turnaround time, and a definitive diagnosis of the causative species is not always possible [1,2].

In the management of IFIs, different treatment strategies have been adopted, and each has its advantages and disadvantages, including prophylaxis, pre-emptive, empiric, and targeted therapy. In terms of prophylaxis, the preventive administration of antifungal therapy to patients at high risk of IFI without attributable signs and symptoms [3] has definitely conferred a significant survival benefit and reduced the incidence of infection in certain patient/treatment settings [4]. In this context, azole prophylaxis has become the standard of care in some patient settings, e.g., hematopoietic stem cell transplant recipients (HSCT), but is often restricted to high-risk patients in an ICU setting [4]. Pre-emptive management relies on the identification of patients most at risk and the utilization of sensitive rapid diagnostic techniques [4]. It subjects fewer patients to toxic and expensive antifungal treatments, but it is difficult to implement due to a lack of sensitive and rapid diagnostic tools [3], while currently, no consensus definition of pre-emptive therapy exists [4]. The initiation of antifungal treatment in patients at high risk of IFIs with established clinical signs and symptoms but without microbiological documentation in the context of empiric therapy is the most common entity encountered [3]. It considers pathogens not covered by drugs previously used in prophylaxis and is often initiated in neutropenic patients with persistent or relapsing fever [4]. Empiric therapy is commonly initiated without knowledge of the susceptibility of fungal strains to selected treatment, but hospitals should always be aware of local resistance rates [5]. When finally, the identification of the causative pathogen occurs, targeted therapy takes place with the initiation of specific antifungal treatment [3]. However, retrospective studies have shown that delayed antifungal treatment of blood-stream infection leads to increased mortality [6,7,8]. In this context, when IFI is suspected but confirmative diagnosis has not yet been possible, the spectrum of activity of antifungal treatment is an important consideration [9], since treatment delay might enhance mortality in this patient population [10].

## 2. Amphotericin B Past and Present

Amphotericin B (AmB) was first introduced in the late 1950s, with polyenes representing the oldest family of antifungal drugs. It represented a suitable antifungal due to its broad spectrum of activity, low resistance rate, and good clinical and pharmacological action. However, AmB has some side effects, such as nephrotoxicity and infusion reactions, which limit its use. To overcome these, new formulations of AmB were developed, including AmB lipid complex, Liposomal AmB, and AmB colloidal dispersion, which have now been discontinued due to a high rate of infusion-related events [11,12,13].

### 2.1. Mechanism of Action

AmB deoxycholate has a micellar structure composed of a colloidal dispersion of amphotericin B with deoxycholate salt in an aqueous glucose solution [11]. The hydrophobic part of the molecule binds to ergosterol in the cytoplasmic membrane of fungi, forming pores and channels in the plasma membrane that allow the extravasation of electrolytes from the intracellular medium, causing cell death. Conventional AmB has a broad spectrum of antifungal activity, but it can also bind to cholesterol in mammalian cell membranes, which can lead to AEs such as nephrotoxicity, a common side effect associated with conventional AmB [14]. On the other hand, in the AmB lipid complex, AmB is delivered via a multi-lamellar ribbon-shaped suspension complex with dimyristoylphosphatidylcholine (DMPC) and dimyristoylphosphatidylglycerol (DMPG). Several mechanisms of action may be involved in the release of AmB, such as hydrolysis using host tissue-derived phospholipases [15]. Incorporation in a lipid complex can actually substantially affect the functional properties of the drug [11]. Last, in liposomal AmB, the liposome is composed of phospholipids, which are stable at mammalian body temperature and incorporate AmB securely into the liposomal bilayer [15]. Liposome size of 60–80 nm provides slower drug absorption by macrophages of RES cells and drug release from the liposome, and it does not allow penetration into distal renal tubules, reducing nephrotoxicity [16]. L-AmB can bind to fungal cell walls, where the liposome is disrupted and released, transferring through the cell wall and binding to ergosterol in the fungal cell membrane, resulting in pore formation, electrolyte release, and fungal cell death [11,15]. The affinity of AmB to the fungal ergosterol is greater than that of mammalian cholesterol, ensuring the drug is only released in the presence of fungal cells [11]. In pre-clinical studies, L-AmB’s mechanism of action resulted in potent in vitro fungicidal activity, while the integrity of the liposome was maintained in the presence of mammalian cells, reducing its toxicity [15].

### 2.2. Pharmacokinetic Parameters

There are biochemical and pharmacokinetic differences between different AmB formulations, while pharmacokinetic parameters suggest that AmB remains associated with the liposome structure while in circulation when delivered as L-AmB. The stability of L-AmB, small size of the particles, and targeting of L-AmB to fungal cell walls facilitate penetration of the liposomes into different tissues. Since the liposomes are less than 100 nm in size, they will initially bypass uptake by the macrophages in the reticuloendothelial (RES) tissues. Over the next 24 h, the circulating liposomes will be slowly taken up by the macrophages and can be found in the highest concentrations in the liver and spleen, resulting in distribution into non-RES tissues of the lungs and kidneys, localization in the epithelial lining fluid (ELF) and alveolar macrophages of the lungs, distal tubules of the kidneys, and macrophages of the liver and spleen, as well as minimal distribution into the brain. Hence, L-AmB administered intravenously distributes to tissues frequently infected by fungi at levels above the minimum inhibitory concentration for many fungi. Moreover, its clinically achievable AUC values are associated with near-complete suppression of galactomannan and (1→3) β-D-glucan levels, which represent markers of therapeutic response in invasive pulmonary aspergillosis [17]. In addition, liposomes change how AmB interacts with the host immune system and, in preclinical models, engender more favorable antifungal effector mechanisms in the setting of excessive PMN-mediated damage to the lung. The immunomodulatory effects of liposomes in neutrophils were confirmed using L-AmB as well as the empty (non-drug-containing) liposome [18]. The proinflammatory properties of AmB may be detrimental in fungal diseases with a component of inflammatory pathology [18]. 

### 2.3. Safety and Interactions

L-AmB presents reduced renal clearance due to intact liposomes (no dissociated AmB) [19] and exhibits a less nephrotoxic profile than free AmB [15]. The pharmacokinetic differences between L-AmB and ABLC also result in different safety profiles. In comparative trials, more patients discontinued ABLC than L-AmB treatment due to toxicity [20]. Patients treated with L-AmB experienced less nephrotoxicity and fewer infusion-related reactions than patients treated with cAmB or ABLC [21]. In a retrospective analysis of patients with renal insufficiency, previous toxicity, or exposure to cAmB receiving ABLC (*n* = 222) or L-AmB (*n* = 105), L-AmB was associated with less nephrotoxicity than ABLC in patients at increased risk of nephrotoxicity [22]. In a randomized, double-blind trial of cAmB (0.7 mg/kg/day, *n* = 87) or L-AmB (3 mg/kg/day, *n* = 86; or 6 mg/kg/day, *n* = 94), L-AmB provided an equally efficacious alternative to cAmB in patients with AIDS and acute cryptococcal meningitis and, at a dosage of 3 mg/kg/day, was accompanied by significantly fewer adverse events [23]. Similarly, in a randomized multicentre study comparing L-AmB (5 mg/kg/day, *n* = 32) with cAmB (1 mg/kg/day, *n* = 34), in neutropenic patients with documented or suspected IFIs, L-AmB (5 mg/kg/day) was superior to cAmB (1 mg/kg/day) with respect to efficacy and safety profile [24]. L-AmB also had significantly fewer adverse events in adults and children than cAmB [25]. Even though nephrotoxicity and electrolyte abnormalities were similar in both L-AmB and ABLC, rigors and febrile episodes were more common with ABLC [26]. Comparative studies with other antifungals did not confirm these results [27,28], even though real-world data showed that L-AmB was associated with better outcomes than other formulations, including severe nephrotoxicity and overall mortality [22,29].

L-AmB can be an option for the treatment of fungal infections in critically ill patients, independent of renal function at the initiation of treatment. L-AmB has been administered to a large number of patients with pre-existing renal impairment at starting doses ranging from 1–3 mg/kg/day in clinical trials, and no adjustment in dose or frequency of administration was required. In a recent report, Alavarez-Lerma et al. administered L-AmB as first-line treatment in 68.8% of critically ill patients with elevated creatine and in 52.8% with normal creatine [30]. In patients with renal function impairment at the start of L-AmB treatment, serum creatinine concentration showed a median decrease of 1.08 mg/dL (44.3%), as compared with baseline (*p* < 0.001). Thirteen (12.3%) patients with normal renal function at the start of L-AmB treatment had a median increase of serum creatinine by 0.07 mg/dL (14.9%, *p* < 0.001), nine of whom received concomitant treatment with one or more nephrotoxic drugs. There was no statistical difference in mortality rates between patients with normal and impaired renal function at the initiation of L-AmB treatment [30]. 

Over the years, a number of reports have explored the benefits of different multifunctional nephroprotective agents, including bicarbonate and normal saline, since AmB exerts its nephrotoxicity via different pathways [31,32,33,34]. It seems that the combination of sodium bicarbonate and normal saline compared to normal saline alone appears to have no superiority in preventing or attenuating different studied aspects of AmB nephrotoxicity [34]. The potential effect of a decrease does occur at the cost of electrolyte disturbances, mainly potassium; however, requirement of long-term supplementation in these patients is not associated with acute kidney injury [35].

Although infusion-related reactions are not usually serious, consideration should be given to precautionary measures for the prevention or treatment of these reactions in patients who receive L-AmB. Reports have shown that there is a significant decrease in all infusion-related reactions in the L-AmB vs. the cAmB group [36], although this is not the case compared to non-AmB regimens [27,28,37]. Slower infusion rates (over 2 h) or routine doses of diphenhydramine, paracetamol, pethidine, and/or hydrocortisone have been reported as successful in the prevention or treatment of infusion-related reactions. In cases of allergic-type reactions, administration of a test dose may be advised in some countries before a new course of treatment. On the other hand, if a severe allergic or anaphylactic/anaphylactoid reaction occurs, the infusion should be immediately discontinued, and the patient should not receive further infusions of L-AmB. Lipid formulations of AmB may be associated with signs of drug-induced lung injury (DILI), but the magnitude and severity of hepatic adverse effects as assessed by drug discontinuation seem to be mild [38].

On top of that, one should take a note of caution when using different liposomal formulations of AmB. Alterations in the size, structure, or composition of the liposomes impact the efficacy and toxicity of the formulation [39]. Hence, it has been reported that copy forms of L-AmB do not have similar efficacy and toxicity profiles [39]. For example, Anfogen^®^ has been reported to be more toxic than L-AmB in a single dose [40], while Lambin^®^ was more toxic than L-AmB based on intravenous dosing in uninfected mice given a single 50 mg/kg dose (80% mortality for Lambin^®^ vs. 0% for L-AmB) [41]. Evaluation of generic formulations of liposomal products should require detailed registration guidance and bioequivalence assessment to ensure similar efficacy, quality, and safety profiles to those presented by the original product, as well as correct drug safety monitoring.

### 2.4. Liposomal Amphotericin B Efficacy and Place in Current Recommendations

A plentiful amount of data has at the moment established L-AmB efficacy in clinical settings. A systematic review and meta-analysis of 23 randomized controlled trials (*n* = 2677) has shown that conventional AmB presents a similar efficacy profile as lipid-based formulations, although the latter are associated with a safer profile [42]. In a randomized, double-blind trial of cAmB (0.7 mg/kg/day, *n* = 87) or L-AmB (3 mg/kg/day, *n* = 86, or 6 mg/kg/day, *n* = 94), L-AmB provided an equally efficacious alternative to cAmB in patients with AIDS and acute cryptococcal meningitis and, at a dosage of 3 mg/kg/day, was accompanied by significantly fewer adverse events [23]. In this context, a further phase 2 study even demonstrated that a single, high dose of L-AmB (10 mg/kg) was non-inferior to 14 daily doses (3 mg/kg) at clearing Cryptococcus from the cerebrospinal fluid and was well tolerated [43]. In line with these findings, a single, high-dose L-AmB (10 mg/kg) given with 5-flucytosine and fluconazole was non-inferior to the current WHO-recommended standard of care for HIV-associated cryptococcal meningitis [44]. 

In neutropenic patients with documented or suspected IFIs, L-AmB 5 mg/kg/day was superior to cAmB 1 mg/kg/day with respect to efficacy and safety [24]. Survival outcomes among neutropenic patients treated with L-AmB or cAmB were similar (93 vs. 90%), but there were fewer proven breakthrough IFIs among patients treated with L-AmB (3.2 vs. 7.8%, *p* = 0.009) [21]. In high-risk patients, initial or intermittent administration of high dose L-AmB has also been reported, offering the benefits of lower treatment costs, improved patient compliance, and reduced toxicity [45]. Infusion of L-AmB doses as high as 10 mg/kg/day may be a good therapeutic option for the management of invasive pulmonary aspergillosis developing in the context of steroid immunosuppression [46]. Despite the absence of significant differences between any of the L-AmB regimens, a trend towards better response rates with the higher loading dose was observed [47]. 

Hence, at the moment, AmB remains the treatment of choice for many serious fungal infections in vulnerable hosts owing to its excellent spectrum of activity and its low resistance rates. L-AmB is recommended as a first-line treatment for several rare mold and rare yeast infections, as well as for the treatment of mucormycosis [48,49,50,51,52,53,54,55,56,57,58]. Table 1 summarizes L-AmB’s current recommendations. 

## 3. Unmet Needs in the Diagnosis and Management of Fungal Infections

### 3.1. Early Diagnosis

Confirmative diagnosis of a suspected fungal infection is challenging due to the low sensitivity of tests and lengthy turnaround times [1]. Culture and microscopy remain the gold standard for proven IFI diagnosis. Mycological culture from blood, sputum, tracheal secretions, or bronchoalveolar lavage fluid is the preferred method to obtain a definitive diagnosis [59], since culture recovers the infecting organism, permits assessment of drug susceptibility, and can detect multiple pathogens. However, the sensitivity and specificity of such methods are limited [1]. Cultures diagnose less than 50% of patients with invasive candidiasis [60] and invasive Fusarium infections [61], while they perform less well for deep-seated infections [62]. Importantly, a 24-h delay in blood culture positivity nearly doubles the risk of death in cancer patients with candidaemia [63]. In every day clinical practice, cultures may take up to four weeks longer than other methods to diagnose invasive candidiasis, and invasive techniques may be required to obtain a sterile site culture [2]. Of note, in the case of Aspergillus, culture yields from blood or bronchoalveolar lavage are low [64], hence positive results are typically unachievable within the early course of invasive aspergillosis [64]. That said, there is a need for more sensitive and targeted diagnostic systems for IFIs, which should directly detect fungal species in clinical specimens [1] and provide more rapid detection. At the moment, there are other methods available for the diagnosis of IFIs, such as PCR, blood β-D-glucan, and galactomannan; however, they too bear disadvantages, and in many cases, they are advised against as sole indicators of IFI for diagnostic decision making [65].

### 3.2. Regimen Spectra and Properties

Treatment, though, may need to begin before a specific species has been identified, meaning that the spectrum of activity of available agents might need to be taken into account when selecting a therapy (Table 2). 

A range of other factors are important when treating empirically suspected IFIs [66], including bioavailability, adverse effects, and interactions with other concomitant treatments. Pharmacokinetic drug-drug interactions are commonly seen across most classes of antifungal agents and more often occur with azoles [67]. Moreover, the development of more oral antifungal options with improved bioavailability and tolerability is desirable, as current intravenous formulations often necessitate hospitalization and can be associated with adverse effects [68,69]. The availability of oral alternatives would be a major advancement, particularly for outpatient management, reducing the burden on healthcare systems and improving patient comfort. Reducing the toxicity of antifungal therapies remains another crucial unmet need. Current antifungal agents are known for their toxicity, especially nephrotoxicity, which can limit their use. Developing formulations with reduced toxicity profiles, such as improved lipid-based formulations or alternative drug delivery systems, is essential to enhancing patient safety.

### 3.3. Increasing Resistance, Emergent Pathogens, and Breakthrough Infections

The emergence of antifungal resistance, particularly in azoles and echinocandins, poses a significant challenge and highlights the need for new antifungal agents with novel mechanisms of action to combat resistant strains effectively [70]. Antifungal resistance is a growing problem associated with a changing epidemiology [71], and broad-spectrum antifungals are needed to fight breakthrough invasive fungal infections in a timely and comprehensive manner. Resistance in *Candida* spp. is increasing and is associated with poorer outcomes [72], mostly caused by non-albicans Candida species such as *C. glabrata* and *C. auris* [73], with reduced susceptibility to first-line antifungals [74]. Azole resistance is a growing phenomenon in *Aspergillus* spp., infections from which are often cryptic [73,75]. Pre-exposure to antifungals can cause an increased proportion of less susceptible species [76]. Moreover, the development of less toxic drugs has led to increased prophylactic use with accompanying increases in drug resistance [73], and this may be associated with breakthrough infections with emerging pathogens that complicate patient management [77].

Breakthrough IFIs develop in the setting of receiving at least seven days of a mold-active antifungal as primary or secondary prophylaxis, as steady-state drug levels are expected by that time [78]. Breakthrough IFIs occur in ~7.5% of patients receiving mold-active systemic antifungal prophylaxis with posaconazole [79] and 10% with low-dose voriconazole [80]. Outcomes of patients with probable/proven breakthrough IFIs are worse than those of patients with a possible breakthrough IFI [81]. A recent study evaluating 397 patients with hematologic malignancy (HM) treated with chemotherapy with persistent fever and suspected IFI showed that there was a significantly lower incidence of proven/probable IFIs in patients treated with empirical antifungal therapy (*n* = 14, 7.4%) than in patients treated with pre-emptive therapy (*n* = 49, 23.7%) (*p* < 0.001) [82]. The rate of deaths attributable to IFIs was significantly lower in subjects treated with empirical antifungal therapy (1 case; 7.1%) than in subjects treated with pre-emptive therapy (11 cases; 22.5%) (*p* = 0.002) [82]. A multicentre, open-label, randomized noninferiority trial, comparing an empirical antifungal strategy (*n* = 150) with a pre-emptive one (*n* = 143), showed that probable or proven IFIs were more common among patients who received pre-emptive treatment than among patients who received empirical treatment (13/143 vs. 4/150, *p* < 0.05) [83]. Overall survival was not lower with pre-emptive treatment (95.1%) than with empirical treatment (97.3%), and the 95% CI for the difference was −5.9% to 1.4% [83].

### 3.4. Respiratory Patient Populations

Moreover, there seems to be a large unmet medical need in a range of respiratory patient populations affected by *Aspergillus* sp. and advanced chronic obstructive pulmonary disease, often leading to hospitalization. Invasive aspergillosis is a serious complication since *Aspergillus* sensitization may worsen symptoms in chronic obstructive pulmonary disease [84]. Moreover, persistent A. fumigatus infection is an independent risk factor for pulmonary exacerbations and hospital admissions causing lung function loss in patients with cystic fibrosis, while 10–57% of cystic fibrosis patients are colonized [85,86]. In addition, Aspergillus increases the risk for obstructive chronic lung allograft dysfunction, the most common cause of death after lung transplantation [87]. To the above populations, there are both COVID-19 and influenza patients, commonly within the ICU [88,89,90]. 

First-line treatment with IV azoles in this indication has the caveat of a narrow therapeutic window and drug-drug interactions (DDIs). In addition, IV antifungals may be contraindicated in patients with multiorgan dysfunction and may not provide sufficient penetration in patients with plaques in the trachea [88,89,90]. The same applies for patients with chronic bronchopulmonary aspergillosis (CBPA), where approximately 3 million patients suffer globally [91]. CBPA is mostly treated with azoles, but 50% of patients relapse [92]. In addition, long-term oral azole antifungal therapy may lead to problems such as inadequate dosing, limited bioavailability, antifungal resistance, and adverse events [91,93]. Of note, up to 20% of azole resistance has been reported. Similarly, in allergic bronchopulmonary aspergillosis (ABPA), undertreatment may lead to pulmonary fibrosis, bronchiectasis, and persistent asthma, along with loss of lung function. Treatment primarily consists of oral or IV azoles with inhaled corticosteroids. However, there are DDIs between itraconazole and corticosteroids and AEs related to long-term use of voriconazole [94,95].

### 3.5. Critically Ill Patients

Similarly, a major challenge in antifungal management lies within the ICU, and there is a difference in the risk of developing an IFI in the ICU depending on the underlying condition [96]. In a retrospective study performed in 23 ICUs in 9 European countries, the cumulative incidence of invasive candidiasis in the ICU was 7.07 episodes per 1000 ICU admissions [97]. In this context, *C. auris* and pan-echinocandin-resistant *C. glabrata* occur in COVID-19 patients and are often fatal [98,99,100]. Patients with neutropenia, hematological malignancy, or allogenic hematopoietic stem cell transplantation (HSCT) are at the highest risk for invasive pulmonary aspergillosis (IPA) in the ICU [101], while new risk groups for IFIs are emerging, including biologic agents, small-molecule kinase inhibitors, CAR-T cells, and COVID-19 [89,96]. As already stated, chronic obstructive pulmonary disease is the most frequent underlying condition for patients without HM diagnosed with IPA in the ICU [102]. In these patients, IPA had a median survival of 29 days, compared with a median survival of 86 days in non-IPA patients [103]. Influenza and COVID-19-related aspergillosis further complicate both diseases, increasing both hospitalization length as well as mortality [104,105]. In this setting, awareness of warning symptoms and signs and a high index of clinical suspicion should be maintained for rhino-orbital-cerebral mucormycosis in patients with COVID-19 [106].

Early diagnosis and treatment are critical to reducing mortality in the ICU, but this is often challenging and suffers from the flaws already discussed above [107]. On top of that, in critically ill patients, the American Thoracic Society suggests against relying solely on the results of serum BDG testing for diagnostic decision-making [65]. In these patients, a range of factors should be taken into consideration in the selection of an appropriate regimen. Patients should be assessed for their clinical stability, when, for example, a fungicidal regimen would be preferred over a fungistatic one. Previous antifungal exposure, local epidemiology, and colonization should be taken into account to assess the risk of infection with less susceptible species, e.g., Candida. Moreover, in these patients with commonly multiorgan failure that require organ support, specific pharmacodynamic and pharmacokinetic properties are to be considered, e.g., if the dose is appropriate for extracorporeal membrane oxygenation (ECMO), renal replacement therapy (RRT), hepatic impairment, etc., and/or therapeutic drug membrane (TDM) is required to ensure effectiveness and prevent toxicity. Patients commonly necessitate a variety of concurrent medications that can interact with antifungals. Last but not least, the site of infection and dissemination is important in order to select the drug with maximal penetration.

## 4. New Antifungals in the Era of Unmet Needs

It has thus become evident that addressing the unmet needs in the treatment of systemic fungal infections is essential to improving patient outcomes and reducing the burden of these potentially life-threatening conditions [68,69]. In addition to addressing these unmet needs, research and development efforts should focus on broad-spectrum antifungal agents that can effectively target a wide range of fungal pathogens, simplifying treatment decisions and improving outcomes, especially in cases where the exact causative agent is unknown. Collaborative efforts among researchers, healthcare providers, pharmaceutical companies, and public health organizations are required to advance antifungal therapies, improve access to treatments, and enhance diagnostic capabilities to meet these challenges in the treatment of systemic fungal infections.

Emerging antifungal agents offer innovative approaches to address these challenges. They have the potential for improved bioavailability, safety profiles, and enhanced efficacy and spectra, particularly against drug-resistant fungal strains. However, at the moment, most of these agents, including ibrexafungerp, otesoconazole, and rezafungin, have only been approved for acute or recurrent episodes of vulvovaginal candidiasis. We are awaiting data from clinical trials pertaining to invasive lethal fungal infections. Of note, mucormycosis is still an issue even in the presence of new regimens that seem to have no potential against the latter. In this context, discussion remains as to whether new oral regimens would find a place among the critically ill, where rapid plasma concentrations are required to ensure the best outcomes. As fungal infections continue to pose a growing threat, the development of these new treatment options is crucial to meeting the evolving needs of patients and healthcare professionals in the fight against fungal diseases. Below, we have provided a brief summary of the new agents (Table 3). 

### 4.1. Rezafungin

Rezafungin, a recently approved antifungal agent, derives its effectiveness from a mechanism of action similar to echinocandins, targeting specific components within fungal cells. This novel antifungal inhibits the synthesis of fungal cell walls, which are vital for maintaining the structure of fungal cells. More precisely, rezafungin focuses on 1,3-β-d-glucan synthase, a key enzyme responsible for producing the essential 1,3-β-d-glucan in the fungal cell wall [108,109]. By disrupting 1,3-β-d-glucan synthase, rezafungin hinders the formation of the fungal cell wall, ultimately weakening and causing the fungal cell to burst. Ιt represents an analogue of anidulafungin designed for increased stability and improved pharmacokinetics [110], allowing for once weekly dosing and front-loading plasma exposure. Its mechanism of action is highly effective against a wide range of fungal species, including Candida—but not *C. glabrata*—and Aspergillus, making rezafungin a promising option for treating various invasive fungal infections. A recent randomized trial (ReSTORE) investigated the once-weekly regimen of rezafungin at doses of 400 mg/200 mg for the treatment of candidemia and invasive candidiasis [111]. Intravenous caspofungin served as the active comparator [111]. Rezafungin demonstrated non-inferiority to caspofungin regarding the primary endpoints of day-14 global cure and 30-day all-cause mortality (23.7%). In comparison with other echinocandins in phase 3 trials, the results indicated improved efficacy and safety, including caspofungin, micafungin, and anidulafungin [112]. An ongoing trial (ReSPECT) has been designed to assess the drug’s role in prophylaxis in allogenic blood and marrow transplant recipients [113]. Considering its properties, it might prove to be a good step-down option for resistant candidiasis, enabling long term therapy [114].

### 4.2. Fosmanogepix

Fosmanogepix, considered a potential game-changer in antifungal treatment, has drawn attention due to its unique way of working and promising lab and animal study results. Initially known as E1210 by Eisai Co. (Tokyo, Japan), this new compound has shown strong effects against various types of fungi [115,116]. Fosmanogepix turns into the active compound manogepix, a groundbreaking kind of antifungal [115]. One of the main ways fosmanogepix works is by stopping the production of glycosylphosphatidylinositol (GPI) through an enzyme called Gwt1. Inhibition of the fungal enzyme Gwt1 facilitates the maturation of glycosylphosphatidylinositol-anchored proteins, thereby affecting fungal cell integrity, growth, and virulence [117]. This drug demonstrates potent activity against most Candida species, with the exception of Candida krusei. Fosmanogepix exhibits equally potent activity against fluconazole-resistant and fluconazole-susceptible Candida strains when compared to fluconazole, itraconazole, voriconazole, amphotericin B, and micafungin [118,119,120]. It also works well against strains of *C. glabrata* that don’t respond to echinocandins [120]. Additionally, it displays potent activity against various filamentous fungi, including Aspergillus fumigatus, and remains active against Fusarium solani and certain black molds. It can also reach tissues such as the central nervous system, making it a good option for treating invasive candidiasis that affects the brain or eyes, where echinocandins might not work as well [121]. In clinical trials, fosmanogepix has completed Phase 1 and Phase 2 tests, showing it’s well-tolerated, absorbed by the body, and promising for treating candidemia. With an ongoing Phase 2 study for Aspergillus and rare molds, as well as a Phase 3 study for candidemia in progress, fosmanogepix could become a valuable addition to antifungal treatments [122]. Given its broad spectrum of activity, fosmanogepix holds promise as a treatment for invasive fungal infections [116]. 

### 4.3. Olorofilm

Olorofim, a novel antifungal classified as an orotomide, emerged from a screening of over 300,000 small molecules [123]. Researchers identified its mechanism of action as the inhibition of dihydroorotate dehydrogenase, a key enzyme in pyrimidine biosynthesis. After its discovery, olorofim underwent pre-clinical studies and phase 1 human trials and is currently undergoing phase 2 clinical trials. Susceptibility tests on various molds have shown broad, although not universal, anti-mold activity [124]. Olorofim exhibits low MICs against *Aspergillus* spp., including azole-resistant strains such as A. terreus and cryptic species of Aspergillus, often lower than existing anti-mold agents [125,126,127]. Its effectiveness extends to Talaromyces, Trichophyton, and Penicillium [128,129]. Notably, olorofim displays potent activity against some challenging molds such as Alternaria, certain Fusarium species, Scedosporium, and Lomentospora, with significantly lower MICs compared to triazoles and amphotericin [130,131]. However, it lacks in vitro activity against yeasts and Mucorales [132]. Early in treatment, olorofim exhibits a fungistatic effect, which transitions to a fungicidal effect with subsequent doses, leading to cell lysis. Mouse models have shown high survival rates against various Aspergillus species, including a model of sinus and pulmonary infection with Aspergillus flavus, where olorofim performed at least as well as posaconazole [133]. Additionally, in a mouse model of CNS Coccidioides infection, olorofim resulted in sustained reductions in fungal burden compared to other antifungal agents [132]. Multiple phase 1 studies, involving single and multiple ascending doses, have been completed, demonstrating that olorofim pharmacokinetics exceed the desired exposure levels. It was well-tolerated in healthy volunteers with a mild transaminase increase and safe short infusion durations that maintained the required exposures. An ongoing phase 2b open-label study is actively recruiting patients with limited treatment options. Several case reports have highlighted successful outcomes against various organisms. Notably, olorofim effectively treated two cases of Lomentospora infections, one localized and the other disseminated, despite the usual resistance of *L. prolificans*. Additionally, it has been used in combination with posaconazole to treat disseminated coccidioidomycosis, even after multiple previous antifungal treatment failures [122]. Preliminary findings from a phase-2b, open-label trial (study 32) indicate that olorofim, in comparison to relevant historical controls or anticipated outcomes for highly active, uncontrolled invasive fungal infections, demonstrates a favorable benefit-risk balance within a clearly defined patient population with restricted or lacking treatment options [134].

### 4.4. Ibrexafungerp

Ibrexafungerp, a novel antifungal, is the first oral glucan synthase inhibitor in the triterpenoid class [135,136]. It inhibits β-1,3-glucan synthase, similar to echinocandins but with some differences in the binding site [136]. It maintains activity against echinocandin-resistant *Candida* spp., minimally affected by FKS mutations [137]. In vitro studies show significant activity against Candida species, including Aspergillus, Pneumocystis, and other fungi [138]. It lacks activity against Fusarium and Mucorales but retains it against Candida FKS-1 and FKS-2 mutant strains [139]. After two successful trials, VANISH 303 and 306, the FDA approved ibrexafungerp for the treatment of vulvovaginal candidiasis [140]. A continuing study (FURI) is currently assessing the efficacy of ibrexafungerp as a treatment option for patients who either cannot tolerate standard antifungal therapy or have not responded to it. Initial findings from the study demonstrate that oral ibrexafungerp yields a positive therapeutic response in patients facing difficult fungal infections with few treatment alternatives available [141]. More ongoing trials explore its use in combination with voriconazole for IPA and its efficacy in severe fungal infections refractory to standard treatments [142,143]. Two ongoing phase 3 trials are assessing ibrexafungerp’s efficacy in severe fungal infections, including Candida auris, while a large randomized phase 3 trial is planned for comparing ibrexafungerp and fluconazole in candidemia and invasive candidiasis patients after echinocandin induction therapy.

### 4.5. Oteseconazole

Oteseconazole (VT-1161) is a novel antifungal designed for improved selectivity, efficacy, and fewer side effects compared to current azoles. Studies indicate it has over 2000-fold selectivity for fungal CYP51 over the human enzyme, potentially reducing drug interactions and toxicity [144,145]. This tetrazole shows broad activity against Candida species, dermatophytes, certain fungi such as Coccidioides, and some Mucorales. Clinical trials have examined oteseconazole for VVC and onychomycosis. In VVC studies, it significantly reduced recurrent VVC in women, with good tolerability [146,147]. In the onychomycosis study, it showed cure rates ranging from 32% to 42%, well-tolerated by 259 patients [148]. While its role in serious invasive fungal infections is uncertain, oteseconazole’s oral formulation, potential for fewer drug interactions, and lower toxicity make it promising. However, it should not be used for VVC in women who plan on becoming pregnant either during use or for several months afterwards because of the long half life and teratogenicity. At the moment, it’s under FDA consideration for recurrent VVC treatment but lacks ongoing studies for IFIs.

### 4.6. Encochleated Amphotericin B

Encochleated AmB (C-AmB) is an innovative treatment for severe fungal infections, aiming to overcome the limitations of traditional intravenous amphotericin B. The intended objective of the new agent is to target delivery, minimize toxicity and improve efficacy. Thus, C-AmB is designed as an orally administered lipid nanocrystal with a unique solid lipid and calcium-based structure [149]. This structure not only protects the drug in the stomach but also allows targeted delivery to specific cells, reducing the typical toxicities associated with AmB. In vitro studies confirm that C-AmB retains AmB’s broad-spectrum antifungal activity and remains effective against various fungal pathogens. Preclinical trials in mice with candidiasis, aspergillosis, and cryptococcosis show good tolerability and effective drug levels in vital organs, including the brain, liver, and spleen [150]. Human clinical trials, including phase 1 and ongoing phase 2 trials in cryptococcal meningitis in HIV patients, indicate that C-AmB is generally well-tolerated, even at higher doses, without traditional amphotericin B side effects such as hyperkalemia, anemia, and kidney issues. This suggests C-AmB could offer a safer and more convenient treatment option [151]. The future of C-AmB seems promising, with ongoing phase 2 trials evaluating its effectiveness in various patient groups, including those with treatment-resistant fungal infections. While more extensive clinical trials are needed to establish its role in treating severe systemic fungal infections, current data strongly suggest its potential to make a significant impact, particularly in cases where existing treatments fall short. Nonetheless, one should consider the fact that C-AmB has miniscule blood levels in humans and, at the moment, is only used in clinical trials along with an effective agent such as fluconazole or flucytosine. It remains to be discussed how soon C-AmB will reach clinical practice in developed countries, taking into consideration the fact that patient disease severity and the adequacy of resources allow for iv administration.

## 5. Amphotericin B Future Perspectives

Even in the presence of developing new agents, L-AmB emerges as a pivotal component in this evolving scenario, presenting itself as a vital choice for managing these infections. It offers wide-ranging antifungal coverage, exhibits low susceptibility to acquired resistance, and presents minimal potential for drug interactions (Table 4) [136].

**Table 4:** New anti-fungal agents’ spectra.

	AmphotericinB	Rezafungin	Fosmanogepix	Olorofim	Ibrexafungerp	Oteseconazole
*Candida albicans*						
*Candida glabrata*						
*Candida parapsilosis*						
*Candida tropicalis*						
*Candida krusei*						
*Candida lusitaniae*						
*Aspergillus fumigatus*						
*Cryptococcus neoformans*						
*Mucorales*						
*Fusarium* spp.						
*Scedosporium* spp.						
*Blastomyces dermatitidis*						
*Coccidioides immitis*						
*Histoplasma capsulatum*						
		No data			Moderate activity	
		No/Minor activity			Strong activity	

### 5.1. Spectrum and Timely Initiation

Even in the presence of developing new agents, L-AmB emerges as a pivotal component in this evolving scenario, presenting itself as a vital choice for managing these infections. It offers a wide-ranging antifungal coverage, exhibits low susceptibility to acquired resistance, and presents minimal potential for drug interactions (Table 4) [152]. 

In this context, identifying susceptible patients or those who are clinically suspected of having a fungal infection, along with early empiric therapy or prophylaxis is of paramount importance, to decrease the mortality and morbidity associated with IFIs [107]. Data from a prospective surveillance study of candidaemia and a retrospective cohort showed that delay and choice of antifungal treatment are associated with poor clinical outcomes and mortality, respectively [153,154]. The key decision in the early treatment of a suspected IFI is the selection of an effective, broad-spectrum antifungal agent [155]. In a retrospective analysis of 121 consecutive Candida bloodstream infections, empiric antifungal therapy was protective against 28-day mortality (OR 0.369; *p* = 0.035). The overall crude mortality was 28.1% and significantly reduced with appropriate empiric antifungal therapy administered within five days (*p* = 0.006) [8]. Similarly, Garey et al. showed that increased time until fluconazole initiation was an independent predictor of mortality (OR, 1.42; *p* < 0.05) in a retrospective study of 230 hospitalized patients with candidemia [6]. Particularly in the case of mucormycosis, delayed AmB-based therapy resulted in a 2-fold increase in mortality rate at 12 weeks after diagnosis, compared with early treatment (82.9% vs. 48.6%) [156]. In a retrospective, subgroup cohort analysis of the AmBiLoad trial, where 107 of 201 patients received 3 mg/kg QD L-AmB for invasive mold infection, survival rates at 12 weeks for possible vs. probable⁄proven cases in the 3 mg/kg group were 82% vs. 58% (*p* = 0.006), respectively [157]. As possible invasive mold disease (IMD), probably reflects an early stage of disease, a better outcome might be expected when treatment with L-AmB is started at an early suspicion of IMD. In line with these findings, a retrospective study of 141 patients with septic shock treated with L-AmB, stratified according to L-AmB treatment initiation either at septic shock onset (early L-AmB group) or after the onset (delayed L-AmB group), was conducted to determine their survival rates [158]. The septic shock cessation period was shorter in the early L-AmB group than in the delayed L-AmB group (7.0 ± 7.0 days vs. 16.5 ± 15.4 days, *p* < 0.001) [158]. Considering other agents, 837 patients were enrolled in a randomized, international, multicentre trial and received empirical voriconazole or L-AmB treatment. Non-inferiority was predefined as a difference in success rates, i.e., absence of a breakthrough fungal infection, survival for seven days beyond the end of therapy, not premature therapy discontinuation, resolution of fever during the period of neutropenia, and successful treatment for any base-line fungal infection, between voriconazole and L-AmB of no more than 10 percentage points [27]. Voriconazole did not fulfill the protocol-defined criteria for noninferiority to L-AmB with respect to the overall response to empirical therapy [27]. Voriconazole patients had a lower rate of serum creatinine increase to >1.5× baseline (*p* < 0.001) compared to L-AmB; however, the rates of increases to >2× baseline were similar in both groups [27]. On the contrary, in another randomized, double-blind, multinational trial of 1095 patients receiving empirical caspofungin (*n* = 556) or L-AmB (*n* = 539) treatment, caspofungin fulfilled the statistical criteria for non-inferiority to L-AmB for empirical antifungal therapy in patients with persistent fever and neutropenia [28]. A post-hoc subgroup analysis of the AmBiLoad trial also indicated that earlier treatment with L-AmB for IMD may decrease mortality vs. waiting until probably/proven diagnosis [157]. These results come in line with a retrospective study of patients with septic shock that showed reduced time to septic shock cessation with early L-AmB compared to the delayed L-AmB group [158].

### 5.2. Increasing Resistance, Emergent Pathogens, and Breakthrough Infections

As already discussed above, it has become evident that fever-driven antifungal treatment decreased IFI-attributable mortality in neutropenic febrile patients with HM compared to initiating therapy after additional laboratory tests or radiographic signs [82]. Epidemiological surveys that examine local and regional resistance trends can be used to guide treatment strategies, while prior antifungal treatment should raise awareness of possible resistance in patients failing therapy. Physicians should keep in mind that azole-resistant *Candida* spp. can be selected even without extended treatment times [159]. US guidance states that azoles should not be used in hematology patients if they have previously received the drug prophylactically [55]. In this context, acquired resistance to AmB is uncommon despite its multiple decades of clinical use, while AmB demonstrates activity against an array of yeast and filamentous fungal pathogens [9]. Liposomal AmB is recommended for suspected resistant Aspergillus and breakthrough fungal infections after azoles [58], while Liposomal AmB is the first treatment choice for mucormycosis [50].

### 5.3. Respiratory Patient Populations

As already stated, at the moment, there is a large unmet medical need in a range of respiratory patient populations affected by aspergillus. Compared with standard-of-care treatment options, inhaled use of L-AmB may provide a number of benefits, including (a) use in patients with hepatic impairment, (b) good tolerance of nebulized delivery, (c) good drug concentrations at site of infection (with lower doses and topical use), (d) decrease in drug toxicity, (e) limited passage into blood plasma, with almost undetectable serum concentrations, (f) potential ways to cope with the emergence of triazole-resistant Aspergillus, (g) potential for both neutropenic hematological patients and non-neutropenic ICU patients at risk, and (h) easier management of pharmacokinetics and avoidance of DDI and TDM issues associated with some azoles [160,161]. In a small proof-of-concept study, inhaled prophylactic L-AmB reduced the likelihood of CAPA development in 78 mechanically ventilated COVID-19 patients [162]. Long-term administration of prophylaxis with nebulized L-AmB was tolerable and prevented *Aspergillus* spp. infection in lung transplant recipients, but resistance to amphotericin B increased over the study period [163]. In a randomized, placebo-controlled trial of 271 patients (407 neutropenic episodes) with hematological disease with expected neutropenia for ≥10 days, prophylactic inhalation of L-AmB significantly reduced the incidence of IPA from 13.6% to 4.3% (*p* = 0.005) [164]. No serious drug-related adverse events were recorded, but coughing occurred more frequently with L-AmB than placebo. In ABPA patients, maintenance therapy using nebulized liposomal AmB did not reduce the risk of severe clinical exacerbation but prolonged the time until the first severe clinical exacerbation [165]. A retrospective study comparing the safety and tolerability of nebulized cAmB or L-AmB in 38 consecutive lung transplant recipients showed that inhaled cAmB and L-AmB were safe and well tolerated over a large number of medication exposures [166]. No significant systemic absorption of L-AmB was detected, and no effect was observed on respiratory function [167,168]. However, although several studies have been published using nebulized L-AmB, available data are inconclusive regarding the efficacy of this use in prophylaxis and treatment of pulmonary IFIs due to a lack of standardization of administration procedures (dose, frequency, delivery device).

### 5.4. Critically Ill Patients

Critically ill patients in the ICU are particularly vulnerable to IFIs due to their complex medical and surgical problems, including disruption of natural barriers to infection, multiple invasive procedures, wide use of devices, and prolonged antibiotic therapies [169,170]. The ICU team faces the challenge of IFIs in both neutropenic and non-neutropenic patients [169,170]. The identity of the specific pathogen is rarely known, so an empirical approach to therapy is often employed, while the choice of antifungal agent is often dependent on the clinical presentation of the patient and the likelihood of infection with a particular organism [171]. Due to its broad activity, AmB is recommended for empirical therapy in certain patients, since early L-AmB administration at septic shock onset may be associated with early shock cessation [158].

However, in this setting, a range of factors should be considered when making the decision to use it, as already discussed [171]. A common concern in these patients is the degree of renal dysfunction that often forbids the use of certain regimens. L-AmB has a less nephrotoxic profile than free amphotericin B and has been shown to be the least toxic lipid formulation in clinical studies. A study of 122 patients in the ICU compared the outcomes of using L-AmB in patients with and without renal impairment [30]. In patients with renal impairment at the start of L-AmB treatment, serum creatinine concentration showed a median decrease of 1.08 mg/dL (44.3%) as compared with baseline (*p* < 0.001), while there was no statistical difference in mortality rates between patients with normal and impaired renal function at the initiation of L-AmB treatment [30]. Moreover, there were no differences in the clinical response (61.1% vs. 56.6%, *p* = 0.953) or microbiological eradication rate (74.1% vs. 64.6%, *p* = 0.382) in patients with or without RRT [30]. Similarly, in a later retrospective, multicentre, observational study including 900 cases, the average daily (*p* = 0.559) and cumulative (*p* = 0.985) dose, treatment duration (*p* = 0.891), and dosing interval (*p* = 0.178) for L-AmB were not significantly different between patients receiving and not receiving RRT [172]. These data suggest that L-AmB can be a treatment option for fungal infections in critically ill patients, irrespective of renal function at the initiation of treatment.

The AmBiDex study evaluated weekly high-dose L-AmB in critically ill septic patients with multiple Candida colonizations [173]. There was no significant increase in serum creatinine levels in patients receiving 10 mg/kg/week L-AmB compared with matched controls [173]. In a phase 3 randomized controlled trial of 537 adult patients receiving at least one dose of micafungin (100 mg/day for patients >40 kg; 2 mg/kg/day for patients ≤40 kg) or L-AmB (3 mg/kg/day), treatment success rates in ICU patients were similar for micafungin (*n* = 120) vs. L-AmB (*n* = 110), 62.5% vs. 66.4%, respectively (*p* = 0.5828) [174].

In the same context, catheter-related infections pose a significant threat within the ICU. Biofilms produced by *Candida* spp. facilitate persistent infection [175,176], while patients with Candida blood stream infections (BSI) have a greater mortality and length of stay in the ICU than patients with Gram-positive or Gram-negative BSI [177]. In an in vitro study, L-AmB was able to destroy >90% *C. albicans* biofilms in 12 h [178]. Especially in the case of *C. parapsilosis,* young biofilm cells are even more susceptible than planktonic cells, making early treatment key [179]. Similarly, L-AmB inhibits and prevents *C. tropicalis* biofilm formation [180]. Of note, L-AmB had the highest activity against biofilms formed by isolates with acquired (*p*-value not significant) or intrinsic (*p* < 0.05) resistance to echinocandins [181].

Regarding commonly encountered aspergillosis, mechanically ventilated COVID-19 patients with prophylactic therapy in the form of inhaled L-AmB had lower rates of CAPA or aspergillus tracheobronchitis compared with patients receiving standard of care [162]. 

## 6. Conclusions

In conclusion, despite the challenges and unmet needs in the treatment of systemic fungal infections that new antifungals tend to address, AmB will continue to be a valuable asset in the years to come. While new antifungal agents are essential to deal with the limitations of current therapies, amphotericin B’s broad-spectrum activity, low incidence of resistance, and efficacy against a wide range of fungal pathogens make it an indispensable option in the antifungal arsenal. With ongoing research and advancements in drug delivery systems, the potential for AmB to be administered in safer and more patient-friendly formulations is promising. As part of a comprehensive strategy for managing fungal infections, AmB will remain a critical tool in saving lives and improving patient outcomes in the battle against systemic fungal diseases.

## Figures and Tables

**Table 1:** L-AmB in Current Recommendations.

Recommending Body	Invasive Mycoses	L-AmB
**Adult Patients**		
ESCMID/ECMM/ERS, 2017 [51]	Empiric therapy	B I
	Invasive pulmonary aspergillosis	B II
	Refractory invasive aspergillosis	B II
ESCMID, 2012 [52]	Candidemia and invasive candidiasis	B I
ECMM, 2019 [50]	Mucormycosis	A II
	Mucormycosis with CNS involvement	A III
ECIL-6, 2017 [53]	Invasive aspergillosis	B I
	Candidemia (overall population)	A I
	Mucormycosis	B II
IDSA, 2016 [54,55]	Candidemia in non-neutropenic patients as an alternative therapy	Strong recommendation, high quality of evidence
	Candidemia in neutropenic patients as an alternative therapy	Strong recommendation, moderate quality of evidence
	Invasive aspergillosis as an alternative therapy	Strong recommendation, moderate quality of evidence
**Paediatric Patients**		
ESCMID/ECMM, 2019 [56]	Invasive aspergillosis	B II
ESCMID, 2012 [57]	Invasive candidiasis in infants	B II
ESCMID/ECMM, 2014 [50]	Mucormycosis	A II
ECIL-8, 2020 [58]	Empiric therapy	A I
	Invasive aspergillosis	B II
	Invasive candidiasis	A II
	Mucormycosis	A II

Note: The strength of recommendation and quality of evidence supporting each recommendation are classified as follows: Grade A: Recommendation for use is strongly supported. Grade B: Recommendation for use is moderately supported. Grade C: Recommendation for use is marginally supported. Grade D: Recommendation against use is supported. Level I: Evidence derived from at least one properly designed randomized controlled trial. Level II: Evidence obtained from at least one well-designed clinical trial without randomization; from cohort or case-controlled analytic studies (preferably from more than one center); from multiple time series; or from significant results of uncontrolled experiments. Level III: Evidence based on the opinions of respected authorities, as informed by clinical experience, descriptive studies, or reports of expert committees.

**Table 2:** Spectrum of available antifungal agents against various fungal pathogens.

	AmB	5FC	FLU	ITR	VOR	POS	ISA	CAS	MICA	ANI
*Candida albicans*	++	++	++	++	++	++	++	++	++	++
*Candida glabrata*	++	++	+	+	++	++	++	+	+	+
*Candida parapsilosis*	++	++	++	++	++	++	++	++	++	++
*Candida tropicalis*	++	++	++	++	++	++	++	++	++	++
*Candida krusei*	++	+	-	+	++	++	++	++	++	++
*Candida lusitaniae*	-	++	++	++	++	++	++	++	++	++
*Aspergillus fumigatus*	++	-	-	+	++	++	++	+	+	+
*Cryptococcus neoformans*	++	++	++	++	++	++	++	-	-	-
*Mucorales*	++	-	-	-	-	++	++	-	-	-
*Fusarium* spp.	+	-	-	+	++	++	++	-	-	-
*Scedosporium* spp.	+	-	-	+	+	+	+	-	-	-
*Blastomyces dermatitidis*	++	-	+	++	++	++	++	-	-	-
*Coccidioides immitis*	++	-	++	++	++	++	++	-	-	-
*Histoplasma capsulatum*	++	-	+	++	++	++	++	-	-	-

AmB: Amphotericin B; 5FC: Flucytosine; FLU: Fluconazole; ITR: Itraconazole; VOR: Voriconazole; POS: Posaconazole; ISA: Isavuconazole; CAS: Caspofungin; MICA: Micafungin; ANI: Anidulafungin; - denotes no/minor activity, + moderate, ++ strong activity; activity varies depending on sensitivity.

**Table 3:** Anti-fungal agents comparison.

	Mechanism of Action	Route of Administration	Spectrum (See Table 4)	Adverse Effects
Amphotericin B	Binds to ergosterol, disrupting fungal cell membrane integrity	IV	Broad spectrum, including *Aspergillus*, *Candida*, and other molds	Nephrotoxicity, infusion-related reactions, electrolyte imbalances
Rezafungin	Inhibits fungal protein synthesis	IV	Broad spectrum, including *Candida* and *Aspergillus*	Gastrointestinal disturbances, hepatotoxicity, QT prolongation
Fosmanogepix	Inhibits glucan synthesis, disrupting fungal cell wall	IV	Broad spectrum, including *Candida* and *Aspergillus*	Gastrointestinal disturbances, increased liver enzymes, QT prolongation
Olorofim	Inhibits fungal ergosterol synthesis	Oral and IV	Broad spectrum, including *Aspergillus* and some molds	Gastrointestinal disturbances, hepatotoxicity, QT prolongation
Ibrexafungerp (formerly SCY-078)	Inhibits fungal beta-glucan synthesis	Oral and IV	Broad spectrum, including *Candida* and some molds	Gastrointestinal disturbances, hepatotoxicity, QT prolongation
Oteseconazole	Inhibits fungal ergosterol synthesis	Oral	Broad spectrum, including *Candida* and *Aspergillus*	Gastrointestinal disturbances, hepatotoxicity, QT prolongation
Encochleated Amphotericin B	Binds to ergosterol, disrupting fungal cell membrane integrity	IV	Broad spectrum, including *Aspergillus*, *Candida*, and other molds	Nephrotoxicity, infusion-related reactions, electrolyte imbalances
